# Interdisciplinary Gerontology in Higher Education: A Case Study From Soup to Nuts

**DOI:** 10.1093/geroni/igab046.265

**Published:** 2021-12-17

**Authors:** Justine McGovern

**Affiliations:** Lehman College, City University of New York, Bronx, New York, United States

## Abstract

Through the lens of a multi-year joint project initiated by faculty in Social Work and Digital Arts at Lehman College, the City University of New York's senior college in the Bronx, NY, this paper provides a guide on how to initiate, implement and evaluate interdisciplinary collaborations in gerontology. The paper also suggests ways to ensure that these collaborations can support tenure and promotion processes, funding initiatives, and pedagogical enhancements. The paper focuses on how to make use of campus resources, including departmental Chairs, research offices, and campus-wide committees to identify appropriate collaborators and funding sources; how to nurture productive interdisciplinary relationships, such as clarifying disciplinary expectations and participants' professional needs; and how to maximize return on the effort for tenure and promotion, such as producing publishable content, identifying appropriate opportunities for interdisciplinary publishing and presenting, advocating for interdisciplinary collaborations, and developing interdisciplinary syllabi, an example of evidence-based high-impact pedagogy.

